# Impact of transportation in freshwater and brackish water on Nile tilapia (*Oreochromis niloticus*) resistance

**DOI:** 10.1186/s12917-024-04194-6

**Published:** 2024-09-06

**Authors:** Mohamed Abd El Aziz Ahmed Abd El-Galil, Hala Ali Alsagheer Abd-Elaal Hassan, Fatma Elzahraa Abd Alhamed Ahmed, Mohamed Abd Allah Mousa, Arafah M. Emam, Ahmed Elsayed Osman

**Affiliations:** 1 Fish Diseases and Management Department, Faculty of Veterinary Medicine, Sohag University, Sohag, 82524 Egypt; 2 Zoology Department, Faculty of Sciences, Sohag University, Sohag, Egypt; 3 Nutrition and Clinical Nutrition Department, Faculty of Veterinary Medicine, Sohag University, Sohag, Egypt; 4 National Institute of Oceanography and Fisheries, NIOF, Cairo, Egypt; 5 Biochemistry Department, Faculty of Veterinary Medicine, Sohag University, Sohag, Egypt

**Keywords:** *O. Niloticus*, Nile tilapia, Transportation, Stress, NaCl, Cortisol, Mucin, Β D, TNF, IL-1β

## Abstract

**Background:**

*Oreochromis niloticus* has great economic value and potential for farming and development. Transportation of fish was done for breeding or trading purpose and it is a challenging aspect of aquaculture. This study aimed to investigate the effect of transportation in freshwater and brackish water on the resistance of *O. niloticus* as well as transportation stress mitigation effect of NaCl. Four equal groups were used; each of 50 fish, the 1st group served as the control (P 1), while the 2nd group (PT 2) was transported in water without salt, the 3rd (PT 3) and 4th (PT 4) groups were transported in water containing 5 gL^− 1^ and 10 gL^− 1^ salt respectively. PT 2, PT 3 and PT 4 were transported for 5 h without any rest or sedative drugs.

**Results:**

The serum cortisol of *O. niloticus* significantly increased at 0 h and then decreased at 12 and 24 h post transportation in the PT 2 group and non-significantly increased at all point times in the PT 3 and PT 4 groups comparing to P 1 group. Mucin2 gene (MUC2) expression was non-significantly up regulated in the PT 2 group and down regulated in the PT 3 and PT 4 groups at 0 h comparing with P 1 group, but at 12 and 24 h it was significantly up regulated in the PT 2, PT 3 and PT 4 groups. The β Defensin-1 (β D1) and 2 (β D2) genes expression was non-significantly down-regulated in the PT 2 group and significantly up regulated in the PT 3 and PT 4 groups at 0 h., while at 12 and 24 h was significantly down regulated in the PT 2 group and non-significantly down regulated in the PT 3 and PT 4 groups, it significantly down regulated in the PT 2 and PT 3 group and non-significantly down regulated in the PT 4 group at 24 h. Non-significant up regulation in interleukin − 1β (IL-1β) gene expression was reported in the PT 2 group and non-significant down regulation in the PT 3 and PT 4 groups at 0 h. However, significant up regulation was recorded in the PT 2, PT 3 and PT 4 groups at 12 and 24 h. The Tumor necrosis factor-alpha (TNF–α) gene expression was non-significantly up regulated in the PT 2 group and non-significantly down regulated in the PT 3 and PT 4 groups at 0 h. However, it was significantly up regulated in the PT 2, PT 3 and PT 4 groups at 12 and 24 h.

**Conclusion:**

The results of this study confirmed the stressful effect of transportation on *O. niloticus* as well as the transportation stress mitigation effect of NaCl.

## Background

The Nile tilapia was initially introduced in developing countries to meet protein demands [1] and it has great economic value and potential for farming and development [2]. Nile tilapia is resistant to environmental conditions and diseases, grows rapidly and adapts to various aquaculture methods, it has relatively low production cost [1]. Improving fish welfare is directly linked to the advancement of fish aquaculture industry. This is because better fish growth is associated to low-stress conditions through lives. Live fish transportation is a common work in aquaculture facilities, but it can activate the stress responses that compromise fish welfare. The impact of transportation stress is influenced by many factors including time, temperature, fish size and health, stocking density, stress level, and packing method [3–5]. Unfortunately, transportation stress also affects water quality as well as fish productivity and survival [6].

Transport stress is one of the factors that raise cortisol level, which activate gluconeogenesis, this process increases glucose levels to provide the energy needed to handle the stress [7]. Increased production of cutaneous mucosal secretions in response to stress has been observed many fish species [8]. Mucin genes were found to be significantly up-regulated in fish after transportation [9, 10]. β-defensins have various activities including antibacterial, antiviral, chemotactic, immune-modulatory, and reproductive regulation. Interleukin-1 (IL-1) is a pro-inflammatory cytokine that plays an important role in fish immunity by activating lymphocytes and phagocytic cells. The tumor necrosis factor-α family is involved in regulating leukocyte homing, proliferation and migration.

Reducing stress during transportation is an essential factor for supporting fish growth and survival rates [11, 12]. Several studies had been conducted on the topic of reducing stress during transportation through using supplementary diet such as probiotics [13, 14], turmeric [15], glycine [16], anesthetics [17, 18] and in the addition of salt to transport water [9, 10, 19]. Adding NaCl to transport water is a common practice in freshwater fish farms to mitigate the adverse effect of transport [20]. Salt is cheap and easy to use in fish farms and it helps alleviate osmoregulation troubles during transport [21–23]. This study aims to investigate the effect of 5 h transportation in freshwater and water containing 5gL^− 1^ and 10gL^− 1^ NaCl on *O. niloticus* resistance as well as the stress mitigation effect of NaCl.

## Materials and methods

### Ethics approval

The protocols of this study were following the ethical consideration of experimental animals and approved by the Veterinary Medical Ethics Research Committee-Faculty of Veterinary Medicine at Sohag University, Egypt, approval number Soh.un.vet/00016 M2.

### Fish

Nile tilapia (*O. niloticus)* (average body weight was 53 ± 3 g) were obtained from Wadi Samhod Tilapia Private Farm in the New Vally Governorate, Egypt.

### Transportation experiment

Nile tilapia was divided into 4 groups, 50 fish for each, the 1st group served as the control group (P1), the 2nd fish group was transported in water without salt (PT2) and the 3rd fish group was transported in water containing 5gL^-1^ Nacl (PT3) and the 4th fish group was transported in water containing 10gL^-1^ Nacl (PT4). The transportation water was obtained directly from the farm pond. The fish were transported for 5 h at stocking density of 26.5gL^-1^ and with continuous aeration; they were transported without sedation or rest. Each fish group was transported in a separate tank with 180 L capacity, containing 100 L of water. Once the fish reached to the Wet Lab. of Fish Diseases and Management Dept., Faculty of Veterinary Medicine, Sohag University, the fish were removed from the transport water to clean freshwater and each group was kept in a separate tank during the sampling time.

### Sampling

Blood and skin samples were collected from the control group (P 1) only pre-transportation, and from the PT 2, PT 3 and PT 4 groups at 0 h, 12 h and 24 h. post transportation, 5 fish from each experimental group were sampled at each determined time point. The fish were anesthetized with MS-222 (150 mgL^-1^) [24] before blood and skin sampling, the blood samples were left to clot at room temperature for sera collection, which was stored at -80 °C until analysis. Skin samples were collected and preserved in Ribonuclic acid *later* (RNA *later)* and stored at -80 °C for gene expression studies [25].

### Cortisol level

The quantity of serum cortisol was determined using ELISA method from a commercial kit (DRG Cortisol ELISA EIA‒1887, Germany). Absorbance readings were taken using a spectrophotometer with a wavelength of 500 nm (HITACHI, U‒2001).

### Gene expression studies

Total RNA was extracted from the skin of *O. niloticus* from control, PT 2, PT 3 and PT 4 using Trizol. 1 µg of total RNA was denatured at 65 °C for 5 min in the presence of 1 µl of oligo-dT17, 1 µl of dNTP (deoxynucleoside triphosphate mix 10 mM (Promega) and RNA/DNA free water (Sigma) in a volume of 13 µl to synthesize cDNA,. The synthesis was carried out using 1 µl of Superscript III reverse transcriptase enzyme (Invitrogen), 5 µl of 5x first strand buffer, 1 µl 0.1 M DTT and water to reach final volume of 25 µl. The mixture was then incubated at 55 °C for 1 h. The resulting cyclic Deoxyribonucleic acid (cDNA) was stored at − 20 °C. The expression of the mucin2 gene (MUC2), antimicrobial peptides (βD1 and 2) and cytokines (IL-1β and TNF-α) was examined before and after transport using RT-qPCR with specific primers (Table 1). For the qPCR, 3 µl of a diluted cDNA template was used following the procedures described by [26]. The relative expression levels of the genes were determined using the Pfaffl method [27] as previously explained [26].

**Table 1:** Showed Oligonucleotide primers were used in SYBR Green real time PCR

Gene	Primer sequence (5’-3’)	Reference
*EF-1α*	CCTTCAACGCTCAGGTCATC	Gröner et al., 2015 [28]
	TGTGGGCAGTGTGGCAATC	
*MUC2*	CAACTGTTTTTGAGACAACTTCAGA	Midhun et al., 2019 [29]
	CTGAAGTGACCGTGGAAGG	
*βD-1*	TTCGCATTGTGTCCTCTGCTCCGTTCGAC	Dong et al., 2015 [30]
	TGAAACAGACAGATCCACATCAAACCCTGA	
*βD-2*	GCTGACAGCAGTGCAAGCTGATGACAC	Taccki et al., 2015 [31]
	GCAAAGCACAGCATCTTAATCTGC	
*TNF alpha*	CCAGAAGCACTAAAGGCGAAGA	Standen et al., 2016 [32]
	CCTTGGCTTTGCTGCTGATC	
*IL-1ß*	AGAGCAGCAATTCAGAGC	Ming et al., 2013 [33]
	GTGCTGATGTACCGT	

### Analysis of the SYBR green Rt-PCR results

Amplification curves and CT values were determined by the stratagene MX3005P software. To estimate the variation of gene expression on the RNA of the different samples, the CT of each sample was compared with that of the control group according to the “ΔΔCt” method [34].

### Statistical analysis

Results are expressed as the mean ± standard error (SE). Data analysis was performed in GraphPad Prism version 5.0 including normality tests. All data were normally distributed. Statistically significant differences were considered when *p* < 0.05. The qPCR measurements were analyzed by T-test to identify statistically significant differences between groups. One-way ANOVA and Tukey post-hoc analysis test were performed to identify statistically significant differences among groups.

## Results

### Cortisol level

**Fig. 1:**
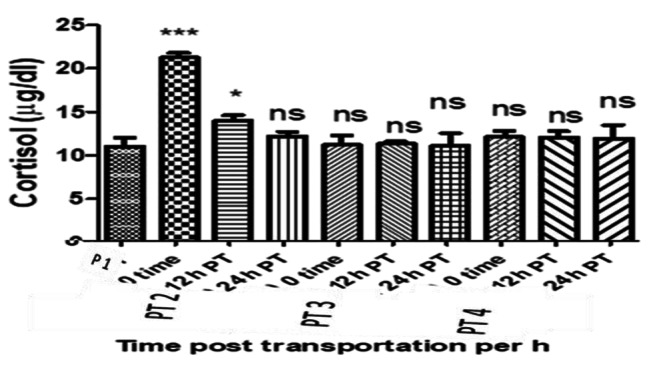
Showed cortisol values in all experimental groups at the sampling times and the significant differences compared to the control group

**Fig. 2:**
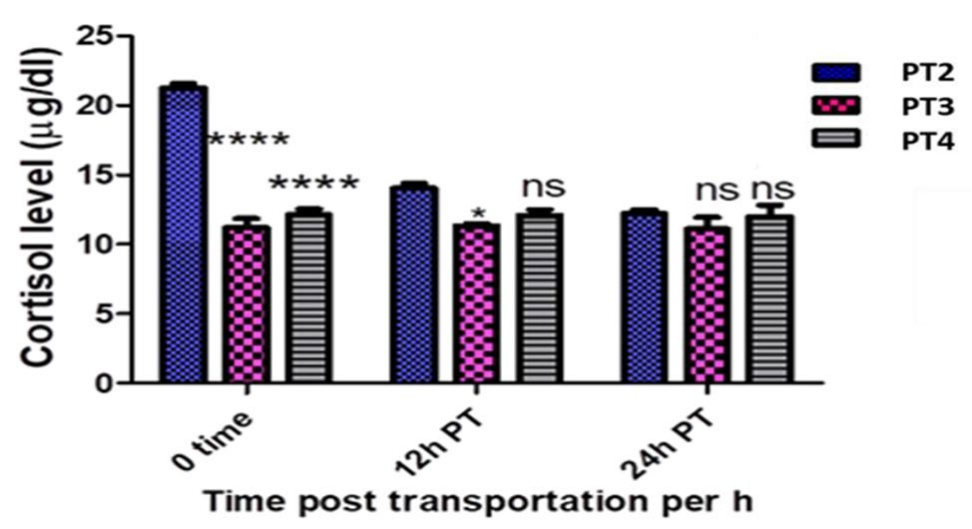
Showed cortisol values in all experimental groups at sampling times as well as the interaction between group-time factors and the significant differences compared to PT2 group

The serum cortisol level of the control *O. niloticus* group (P 1) was 11.07 ± 1.0 µgdl^− 1^. It significantly increased in the PT 2 group at 0 h, decreased greatly but still significantly higher than P 1 group at 12 h and comes back to the basal level without significant difference at 24 h post transportation compared to the control group. However It remains around the basal level and non-significantly increases in the PT 3 and PT 4 groups comparing with the P 1 group at all point times and significantly decreases in the PT 3 and PT 4 groups comparing with PT 2 group at all point times, (Table 2 and Figs. 1 and 2)

**Table 2:** Showed Cortisol values in all experimental groups at the point times and the significant differences compared to the control group

Fish group	Time	Cortisol level (µg/dl) (Mean ± SE)
PT1 (Control group)	Pre-transport	11.02 ± 1.0
PT2 (without Nacl)	0 h post transport	21.27 ± 0.51***
PT3 (5 gL^− 1^ Nacl)		11.70 ± .31^ns^
PT4 (10 gL^− 1^ Nacl)		12.21 ± 0.71^ns^
PT2	12 h post transport	14.03 ± 0.60*
PT3		11.33 ± 0.27^ns^
PT4		12.07 ± 0.72^ns^
PT2	24 h post transport	12.20 ± 0.55^ns^
PT3		11.10 ± 0.46^ns^
PT4		11.96 ± 1.55^ns^

^*^p ≤ 0.05, ^**^p ≤ 0.01, ^***^p ≤ 0.001 and ns = non significant

### Gene’s expression

#### MUC2 gene expression

**Fig. 3:**
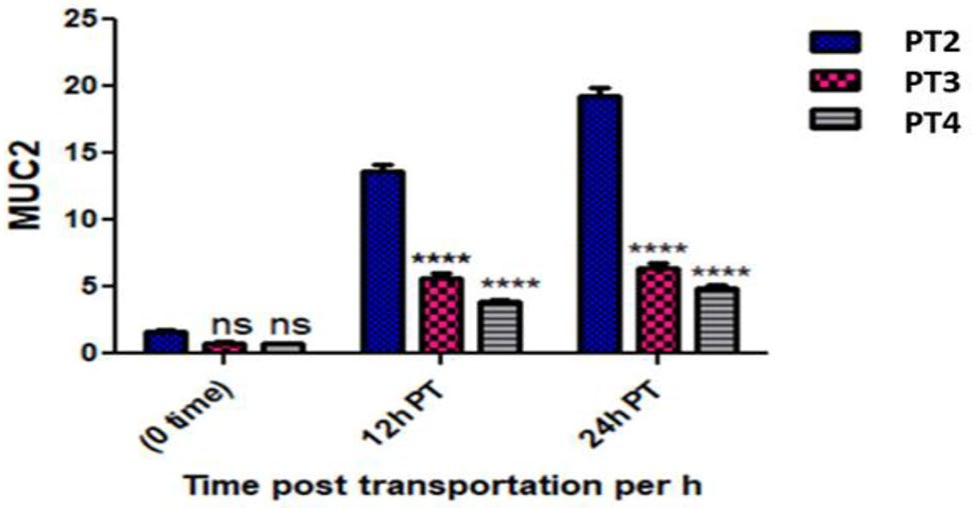
Showed MUC2 gene expression in all experimental groups at the sampling times and the interaction between group-time factors and the significant differences vs. PT2 group

The expression of MUC2 gene *in O. niloticus* non-significantly up regulated in the PT 2 group and non-significantly down regulated in the PT 3 and PT 4 groups at 0 h post transportation comparing with the P 1 group. It significantly up regulated in the PT 2, PT 3 and PT 4 groups at 12 and 24 h post transportation comparing with the P 1 group. However comparing with PT 2 group, it non-significantly down regulated in the PT 3 and PT 4 groups at 0 h post transportation and significantly up regulated in the PT 3 and PT 4 groups at 12 and 24 h. Additionally there was a non-significant up regulation in PT 3 group compared to the PT 4 fish group at 0, 12 and 24 h. (Table 3 and Fig. 3).

**Table 3:** Showed MUC2 gene expression in all experimental groups at the point times and the significant differences compared to the control group

Fish group	Time	EF1α	Muc-2	
		CT	CT	Fold change (Mean ± SE)
P1 (Control group)	Pre-transport	19.36	23.29	-
PT2 (without Nacl)	0 h post transport	19.34	22.62	1.61 ± 0.26^ns^
PT3 (5gL^− 1^ Nacl)		20.87	25.10	0.82 ± 0.04^ns^
PT4 (10gL^− 1^ Nacl)		19.63	24.01	0.74 ± 0.06^ns^
PT2	12 h post transport	19.47	19.63	13.69 ± 0.78***
PT3		20.24	21.67	5.68 ± 0.66***
PT4		20.13	22.09	3.93 ± 0.15***
PT2	24 h post transport	20.08	19.74	19.31 ± 0.94***
PT3		20.66	21.91	6.43 ± 0.68***
PT4		20.72	22.38	4.870.54***

* p ≤ 0.05, ** *p* ≤ 0.01 *** *p* ≤ 0.001 and ns = non-significant

### Antimicrobial peptides (β Defensin– 1 and β defensin − 2)

**Fig. 4:**
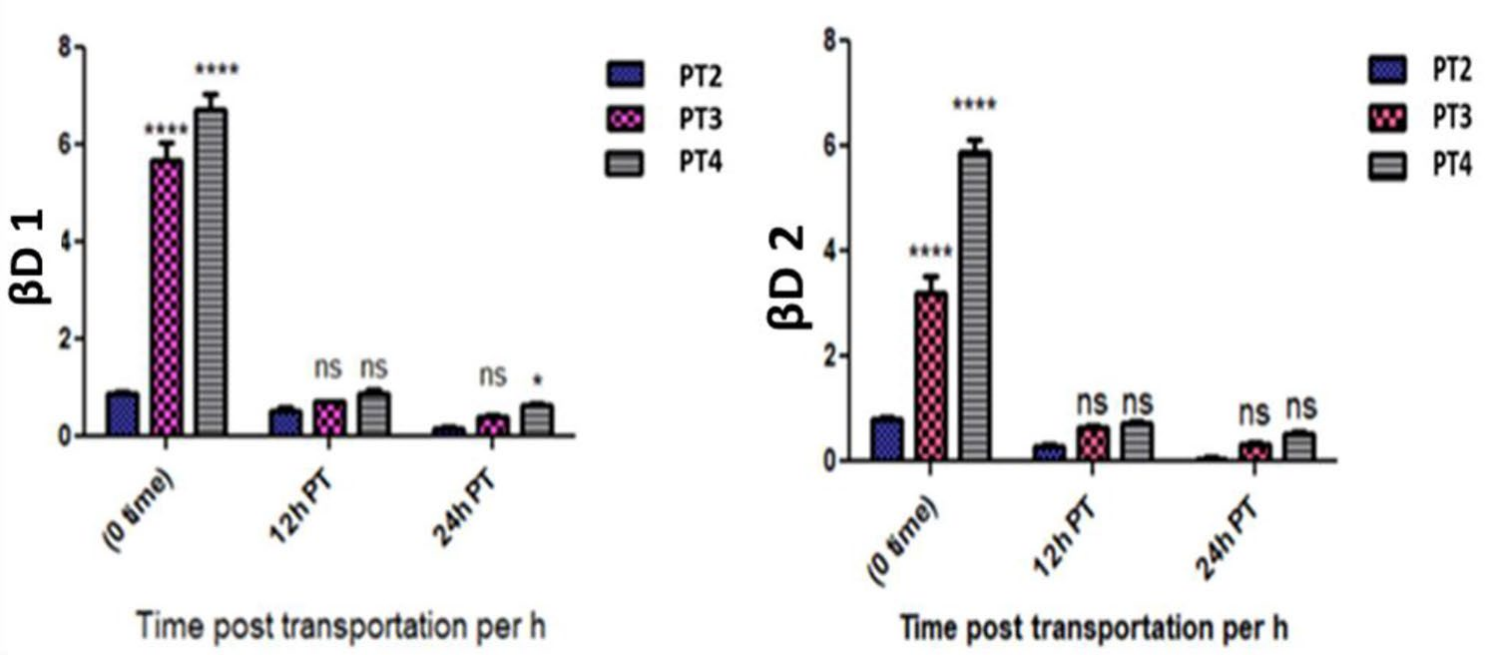
Showed βD-1 and βD-2 genes expression in all experimental groups at the sampling times, the interaction between group-time factors and the significant differences vs. PT2 group

The expression of the antimicrobial peptides βD-1 and 2 genes at 0 h post transportation non-significantly down-regulated in the PT 2 group and dramatically and significantly up regulated in the PT 3 and PT 4 groups comparing with the P 1 control group. At 12 h, they significantly down regulated in the PT 2 group and non-significant down regulation in the PT 3 and PT 4 groups matching with P 1 control group. At 24 h post transportation they significantly down regulated in the PT 2 and PT 3 groups and non-significant down regulation in the PT 4 group comparing with the P 1 group. Matching with the PT 2 group; the βD-1 and 2 significantly up regulated in the PT 3 and PT 4 groups at 0 h post transportation, and they non significantly up regulated in the PT 3 and PT 4 groups at 12 and 24 h post transportation. (Table 4; Fig. 4).

**Table 4:** Showed βD-1 and βD-2 genes expression in all experimental groups at point times and the significant differences compared to the control group

Fish group	Time	EF1α	βD-1		βD-2	
		CT	CT	Fold change (Mean ± SE)	CT	Fold change (Mean ± SE)
P1 - Control group	Pre transport	19.36	21.86	-	20.75	-
PT2 (without Nacl)	0 h post transport	19.34	21.97	0.90 ± 0.03ns	21.05	0.82 ± 0.04ns
PT3 (5 gL^− 1^ Nac)l		20.87	20.86	5.70 ± 0.34***	20.59	3.22 ± 0.55***
PT4 (10 gL^− 1^ Nacl)		19.63	19.38	6.75 ± 0.29***	18.46	5.91 ± 0.38***
PT2	12 h post transport	19.47	22.91	0.53 ± 0.08*	22.63	0.30 ± 0.04*
PT3		20.24	23.23	0.71 ± 0.02ns	22.27	0.64 ± 0.06ns
PT4		20.13	22.80	0.90 ± 0.07ns	22.00	0.72 ± 0.05ns
PT2	24 h post transport	20.08	25.18	0.17 ± 0.05***	25.43	0.06 ± 0.02***
PT3		20.66	24.46	0.41 ± 0.03**	23.60	0.34 ± 0.05*
PT4		20.72	23.90	0.63 ± 0.05ns	23.02	0.53 ± 0.05ns

* p ≤ 0.05, ** *p* ≤ 0.01 *** *p* ≤ 0.001 and ns = non-significant

### Tumor necrosis factor (TNF) gene expression

**Fig. 5:**
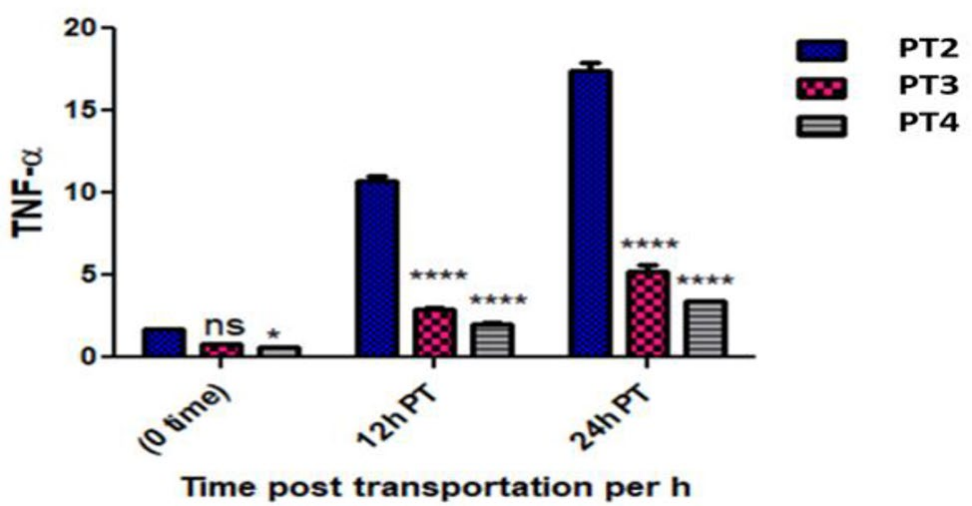
Showed TNF-α measurement in all experimental groups at different times and the interaction between group-time factors and the significant differences vs. PT2 group

The expression of the TNF-alpha gene in *O. niloticus* was non-significantly up regulated in the PT 2 group and non-significantly down regulated in the PT 3 and PT 4 groups compared to the control group at 0 h post transportation. However at 12 and 24 h post transportation; there was a significant up regulation in gene expression in the PT 2, PT 3 and PT 4 groups. Matching with PT 2 group, TNF non significantly down regulated in the PT 3 group and significantly down regulated in the PT 4 group at 0 h post transportation, however at 12 and 24 h post transportation it significantly down regulated in the PY 3 and PT 4 groups. (Table 5; Fig. 5).

**Table 5:** Showed TNF gene expression in all experimental groups at the point times and the significant differences vs. the control group

Fish group	Time	EF1α	TNF alpha	
		CT	CT	Fold change (Mean ± SE)
P1 (Control group)	Pre-transport	19.36	22.15	-
PT2 (without Nacl)	0 h post transport	19.34	21.35	1.73 ± 0.05 ^ns^
PT3 (5gL^− 1^ Nacl)		20.87	23.97	0.81 ± 0.10 ^ns^
PT4 (10gL^− 1^ Nacl)		19.63	23.13	0.61 ± 0.05 ^ns^
PT2	12 h post transport	19.47	18.84	10.71 ± 0.59 ***
PT3		20.24	21.47	2.95 ± 0.20 ***
PT4		20.13	21.90	2.04 ± 0.20 *
PT2	24 h post transport	20.08	18.75	17.40 ± 0.85 ***
PT3		20.66	21.07	5.23 ± 0.61***
PT4		20.72	21.76	3.38 ± 0.09***

* *p* ≤ 0.05, ***p ≤ 0.001 and ns = non-significant

### Interleukin − 1β (IL-1β) gene expression

**Fig. 6:**
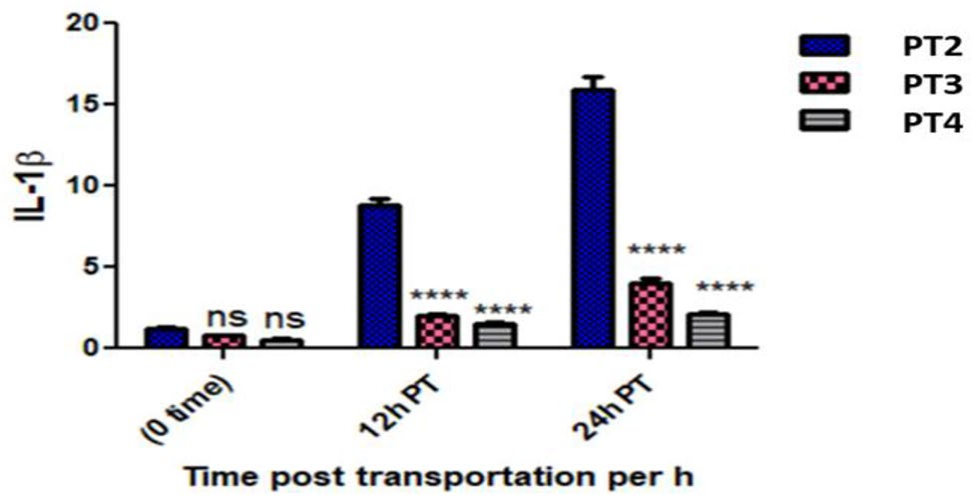
Showed IL-1β gene expression measurements in all experimental groups and the interaction between group-time factors and the significant differences vs. the PT2 group

The results showed that the expression of IL-1β gene in *O. niloticus* was non significantly up-regulated in the PT 2 group and also not significantly down regulated in the PT 3 and PT 4 groups at 0 h post transportation. However, at 12 and 24 h post transportation there was significant up regulated of IL-1β in the PT 2 and PT 4 fish groups group and non-significant up regulation in the PT 3 group comparing with the P 1 group. The IL-1β gene expression significantly up regulated in the PT 2, PT 3 and PT 4 fish groups compared to P 1 fish group at 24 h post transportation. Matching with the PT 2 group; the IL-1β gene expression non significantly down regulated at 0 h post transportation and significantly up regulated at 12 and 24 h post transportation in the PT 3 and PT 4 fish groups (Table 6; Fig. 6).

**Table 6:** Showed IL-1β gene expression in all experimental groups at the sampling times, the interaction between group-time factors and the significant differences vs. the control group

Fish group	Time	EF1α	IL-1β	
		CT	CT	Fold change (Mean ± SE)
P1 (Control group)	Pre-transport	19.36	21.44	-
PT2 (without Nacl)	0 h post transport	19.34	21.06	1.20 ± 0.14 ^ns^
PT3 (5gL^− 1^ Nacl)		20.87	23.32	0.78 ± 0.05 ^ns^
PT4 (10gL^− 1^ Nacl)		19.63	22.62	0.54 ± 0.07 ^ns^
PT2	12 h post transport	19.47	18.41	8.84 ± 0.74***
PT3		20.24	21.28	2.05 ± 0.13*
PT4		20.13	21.66	1.47 ± 0.18^ns^
PT2	24 h post transport	20.08	18.17	15.89 ± 1.30***
PT3		20.66	20.74	4.01 ± 0.46***
PT4		20.72	21.70	2.15 ± 0.04*

* *p* ≤ 0.05, ***p ≤ 0.001, ****p ≤ 0.0001 and ns = non-significant

## Discussion

Many aquaculture operations involve the transportation of live fish from one facility to another or during restocking practices. It has been clarified that the immune response in stressed fish is suppressed [35, 36]. Previous studies on the fish immune response to stress have primarily focused on systemic parameters such as blood cell counts and serum innate immune factors, while neglecting the role of skin immunity. In this study, we investigated the resistance of *O. niloticus* particularly the skin to live transport as well as the stress mitigation effect of salt.

Regarding the serum cortisol, it was significantly increased at 0 h post transport in PT 2 group transported in water without salt compared to both P 1 control group and PT 3 and PT 4 fish groups transported in water containing salt. This elevation may be attributed to the stressful effect of transportation and the importance of cortisol during stress conditions as it elevates blood glucose and stimulate the central nervous system to restore fish body homeostasis [36]. These results are supported by [9] who mentioned that stressed trout had higher cortisol levels than the control group and [10] who recorded that serum cortisol levels in *O. niloticus* significantly increased after 5 h transportation in water without salt. A significant decrease in cortisol level was reported in the PT 3 and PT 4 fish groups compare to with PT 2 group at different sampling times, and similar findings were reported in common carp [12], matrinx˜a, *Brycon amazonicus* [37] and ruho carp, *Labeo rohita* [23]. This may be indicate that salt minimizes the stressful effect of transportation and enhances fish hydro-mineral balance by reducing the osmolality differences between the transporting water and fish body [38, 39]. Cortisol level significantly decreased and recovered to the basal level at 12 and 24 h post transportation in PT 2 fish groups. This result came in line with [40, 41] who found that the cortisol elevation syndrome recovered after 24 h post stress in juvenile tambaqui *colossoma macropomum* and Nile tilapia.

The expression of MUC2 gene *in O. niloticus* non-significantly up regulated in the PT 2 group and non-significantly down regulated in the PT 3 and PT 4 groups at 0 h post transportation comparing with the P 1 group. It significantly up regulated in the PT 2, PT 3 and PT 4 groups at 12 and 24 h post transportation comparing with the P 1 group. However comparing with PT 2 group, it non-significantly down regulated in the PT 3 and PT 4 groups at 0 h post transportation and at 12 and 24 h, it significantly up regulated in the PT 3 and PT 4 groups. Additionally there was a non-significant up regulation in PT 3 group compared to the PT 4 fish group at 0, 12 and 24.

Mucins are important high molecular weight glycoproteins for the physical barrier, mucous viscosity and trapping pathogens in fish [42]. The expression of MUC2 gene in the *O. niloticus* of the PT 2 group was non significantly up-regulated at 0 h and significantly up-regulated at 12 and 24 h post transportation comparing with P 1 group, this up regulation may be attributed the stressful condition of transportation such as confinement, high ammonia level and shacking. This result is consistent with previous studies [9] who reported a significant up-regulation of mucin gene in trout fish post transportation and [10] who stated that the expression of mucin2 gene was significantly up regulated in *O. niloticus* after transportation. MUC2 gene expression of PT 3 and PT 4 groups transported in water containing salt was non significantly down regulated compared with P 1 group at 0 h post transportation. This down regulation may be attributed to the sodium chloride mitigates the transportation stress by decreasing the salinity difference between the fish body and the transporting water as well as controlling mucous secretion on the skin [43]. These results are consistent with [10] who recorded significant down regulated of the MUC2 gene expression in *O. niloticus* transported in water containing 5 gL^-1^ compared with the fish group transported in water without salt. Additionally [8] and [9] reported an increased cutaneous mucosal secretions and significant up-regulation of mucin genes in response to transport stress.

Antimicrobial peptides are a component of the innate immune system of fish and found on the surface layer of epithelial tissues. They act as the first line of defense against various pathogenic invasions. The significant down-regulation of β D-1 and 2 genes expression in the skin of *O. niloticus* transported in water without salt (PT 2 group) at 0, 12 and 24 h post transportation may be attributed to the immune suppressive effect of transportation on skin immunity. Similar down regulation of βD-1 and 2 has been reported in rainbow trout [9] and *O. niloticus* [10] who found that the transportation lead to a significant down regulation of βD -1 and 2 genes expression. The β D -1 and 2 gene expression was up-regulated in the PT 3 and PT 4 groups at 0 h post transportation compared to the PT 2 group, and this may be attributed to the salt mitigated the stress mitigation effect and alleviated the immune suppressive effect of transportation [44]. Moving *O. niloticus* from transporting water containing salt to freshwater down regulated the expression of β D-1 and 2 genes and subsequently suppressed fish immunity at 12 and 24 h post transportation. These results were supported by [12] who found that the addition of 3 gL^− 1^ salt to transportation water for common carp mitigated immunosuppression. The results of our work demonstrate that the expression of β defensin 1 and 2 genes could be used as early response marker to acute transportation stress.

The TNF-α and IL-1β cytokines in teleost fish are powerful pro-inflammatory cytokines released by several immune cells during infection or tissue damage [45]. Overall, transportation stress increases the pro-inflammatory cytokines TNF-α, and IL-1β. The results of this study showed a significant up regulation in IL-1β gene expression of the PT 2 group transported in water without salt at 12 and 24 h post transport and non-significant up regulation at 0 h. This result agrees with the results of [10] who reported up regulation of IL-1β gene expression in *O. niloticus* transported for 5 h in water without salt. The significant up regulation of IL-1β in the PT 2 group indicates that the transported *O. niloticus* in water without salt may be exposed to stressful condition which stimulates IL-1β production along the 24 h investigation time post transportation because the IL-1β acts as an immune and inflammatory response mediator in fish [46]. The IL-1β gene expression in the PT 3 and PT 4 groups down regulated at 0 h post transportation, that may be attributed to the up regulation of β D-1 and 2 in these groups, which have a fascinating ability to suppress the inflammatory response [47]. The IL-1β gene expression up regulated in the PT 3 and PT 4 group at 12 and 24 h post transportation, that may be attributed to the elevated cortisol level [48] and the down regulation of antimicrobial peptides β D-1 and 2 as a result of moving fish from water containing salt to freshwater.

Fish TNF-α acts as regulator and amplifier for acute and chronic inflammation, it is one of the early immune genes that is expressed at the early stage of infection [49]. It has overlapping functions with IL-1β and able to activate macrophages and enhance their microbial killing activity [46, 50, 51]. TNF α gene expression of *O. niloticus* in the PT 2 fish group was significantly up regulated at 12 and 24 h post transport and non-significantly up regulated at 0 h compared with the P 1 group, that indicates the transportation increased the skin inflammatory reaction up to 24 h post transportation [52]. In contrast, TNF-α gene expression was down regulated in the PT 3 and PT 4 *O. niloticus* groups at 0 h post transportation then up regulated at 12 and 24 h post transportation. This may explain the stress mitigation effect of salt during transportation which may extend up to 24 h post transportation.

## Conclusion

The *O. niloticus* group transported in water without salt appeared higher transportation stress effects as evidenced by increased cortisol level and up regulation of Muc2, IL-1B and TNF genes as well as down regulation of β D-1 and 2. However, the addition of sodium chloride to the transportation water had a stress mitigation effect on O. niloticus. This was observed through improvements of fish physiology and mucosal health as well as enhanced skin mucous barrier and immunity; these improvements were more evident in the 5 gL^− 1^ group than 10 gL^− 1^ group. Therefore, it is recommended to use 5gL-1 salt during O. niloticus transportation as it is more beneficial and effective in reducing transportation stress. Further research is required to enhance the well-being of O. niloticus during the transportation process.

## Data Availability

the original data of analysis tests and examination provided within the manuscript are available through the corresponding authar.

## Abbreviations

CT: Cycle Threshold
DNA: Deoxyribonucleic acid
IL-1β: Interleukin-1 β
MS-222: Tricaine methanesulfonate
MUC2: Mucin2
*O. niloticus*: *Oreochromis niloticus*
RNA: Ribonucleic acid
TNF: Tumor Necrosis Factor
βD-1: β Definsin-1
βD-2: β Definsin-2
